# Isolated complete caudate lobectomy for hepatic tumor of the anterior transhepatic approach: surgical approaches and perioperative outcomes

**DOI:** 10.1186/1477-7819-11-197

**Published:** 2013-08-16

**Authors:** Jia-Hua Yang, Jun Gu, Ping Dong, Lei Chen, Wen-Guang Wu, Jia-Sheng Mu, Mao-Lan Li, Xiang-Song Wu, Yang-Lu Zhao, Lin Zhang, Hao Weng, Qian Ding, Qi-Chen Ding, Ying-Bin Liu

**Affiliations:** 1 Department of General Surgery, Xinhua Hospital, School of Medicine, Shanghai Jiaotong University, 1665 Kongjiang Road, Shanghai 200092, China; 2 Shanghai Jiaotong University Xinhua Clinical Medical School, 1665 Kongjiang Road, Shanghai 200092, China; 3 Department of General Surgery, Putuo Hospital, Shanghai University of Traditional Chinese Medicine, Shanghai, China

## Abstract

**Background:**

How to resect the caudate lobe safely is a major challenge to current liver surgery which requires further study.

**Methods:**

Nine cases (6 hepatic cell carcinoma, 2 cavernous hemangioma and 1 intrahepatic cholangiocacinoma) were performed using the anterior transhepatic approach in the isolated complete caudate lobe resection. During the operation, we used the following techniques: the intraoperative routine use of Peng’s multifunction operative dissector (PMOD), inflow and outflow of hepatic blood control, low central venous pressure and selective use of liver hanging maneuver.

**Results:**

There were no perioperative deaths observed after the operation. The median operating time was 230 ± 43.6 minutes, the median intraoperative blood loss was 606.6 ± 266.3 ml and the median length of postoperative hospital stay was 12.6 ± 2.9 days. The incidence of complications was 22.22% (2/9).

**Conclusion:**

PMOD and “curettage and aspiration” technique can be of great help of in the dissection of vessels and parenchyma, clearly making caudate lobe resection safer, easier and faster.

## Background

The caudate lobe, which is generally divided into three regions: the left Spiegel’s portion, the process portion, and the paracaval portion, is located in a complex anatomical position, deep behind the confluence of the main hepatic veins, *porta hepatis* and inferior vena [1]. In other words, it is surrounded by three portae hepatis. The blood supply and biliary drainage of the caudate lobe come from both the left and the right portal triads, called the caudate portal triads (CPT). However, the number of triads may vary. Venous drainage (short hepatic vein) occurs along its posterior aspect directly into the inferior *vena cava* (IVC) through several small branches of variable size and location. Biliary drainage includes small tributaries to the right but occurs predominantly through the left hepatic duct.

**Figure 1:**
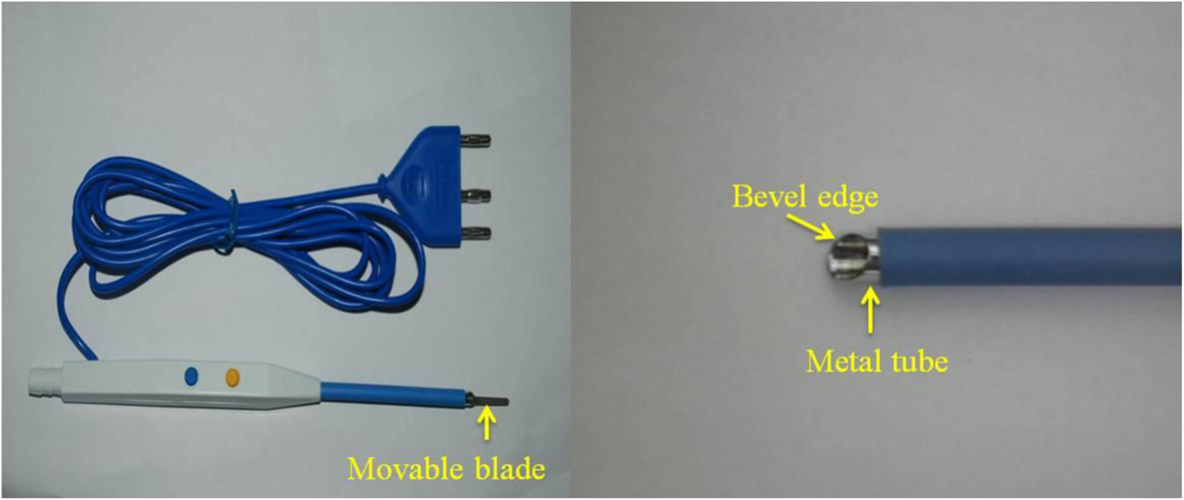
Peng’s multifunction operative dissector (PMOD) and curettage and aspiration dissection technique (CADT).

Due to the deep location and position between the major vascular structures, the caudate lobe has been always considered a forbidden area for hepatic surgery, and its resection is always a challenge for hepatobiliary surgeons. However, with solid knowledge of the anatomical relationship, mastery of the appropriate surgical instrument and thorough experience of performing the operation, the caudate lobectomy can be carried out safely. Isolated complete caudate lobectomy is the most difficult and most complex of the various methods of caudate lobe resection [2]. The anterior transhepatic approach for isolated complete caudate lobe resection has been carried out in our department. In this article, it is proved to be safe, effective and clinically feasible using the curettage and aspiration technique (CADT) and a special instrument, Peng’s multifunction operative dissector (PMOD) (Figure 1).

## Case Presentation

### Patients

Five male and four female patients with a median age of 57 years received isolated hepatic caudate lobectomy between January 2005 and December 2011. Postoperative pathology results identified six patients with hepatocellular carcinoma, two with cavernous hemangioma, and one with intrahepatic cholangiocacinoma. All patients’ liver function was in Child-Pugh class A.

### Surgical procedures

**Figure 2:**
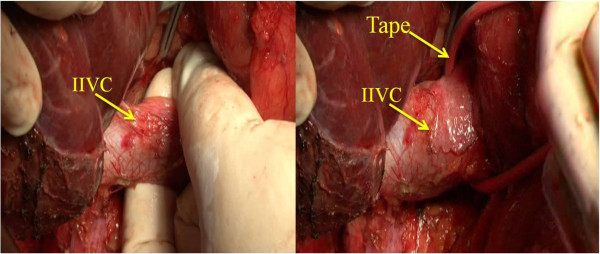
**Opening the retroperitoneum.** The retroperitoneum overlying the infrahepatic inferior vena cava (IIVC) is opened (left). The IIVC is taped (right).

**Figure 3:**
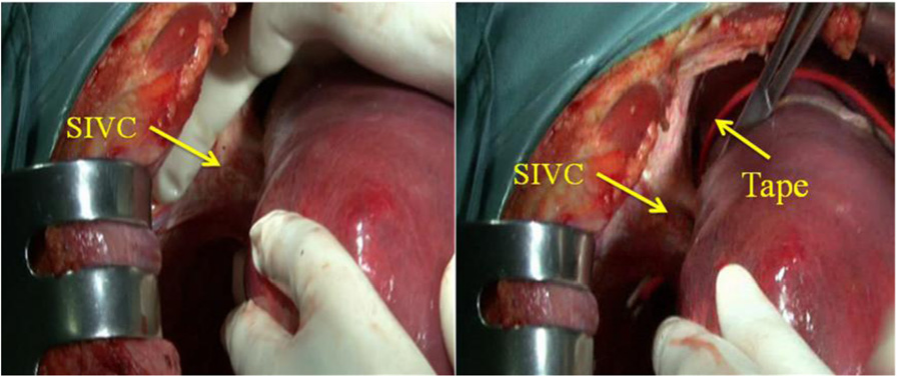
**The tunnel (arrow) was posterior to the suprahepatic inferior vena cava SIVC.** The position of the tunnel is shown (left). A clamp was passed beneath in the channel toward the left side, then a tape was guided to tap the SIVC.

**Figure 4:**
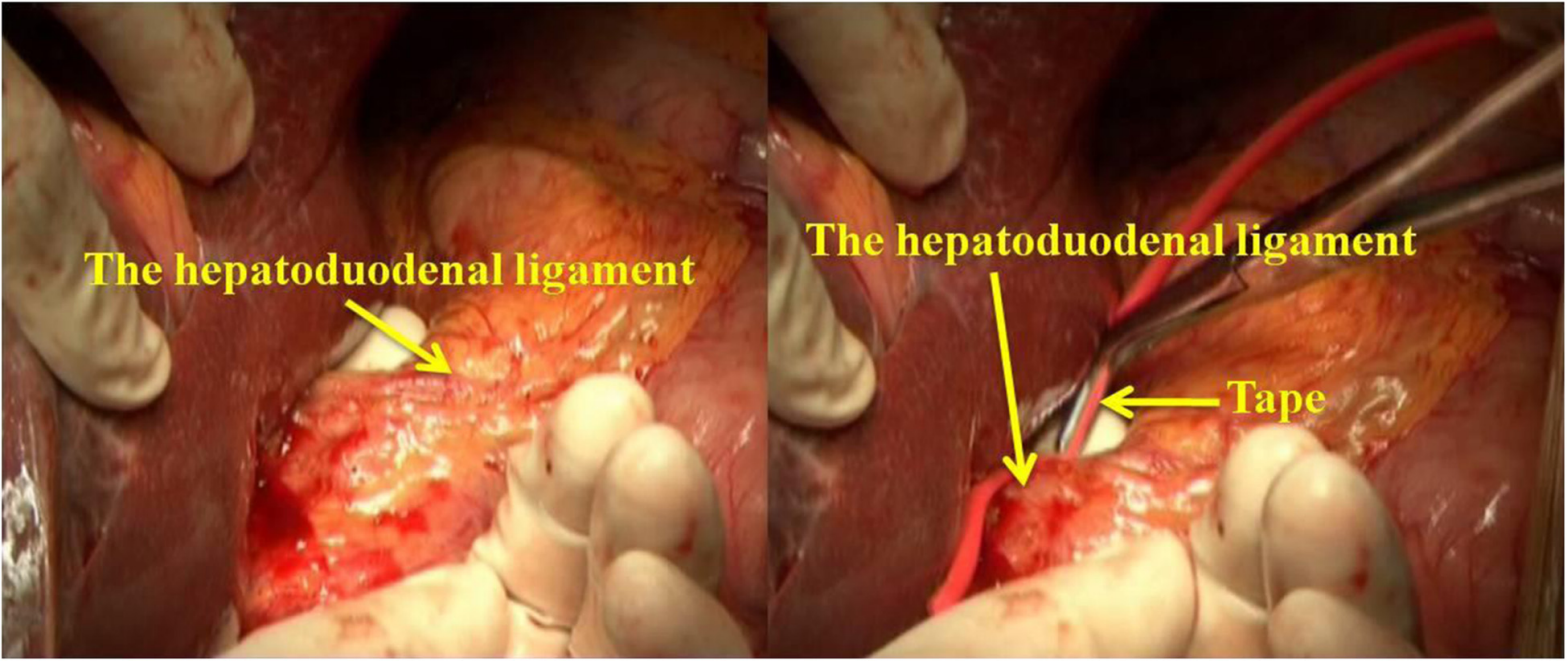
Mobilizing the hepatoduodenal ligament and encircling it with tape.

**Figure 5:**
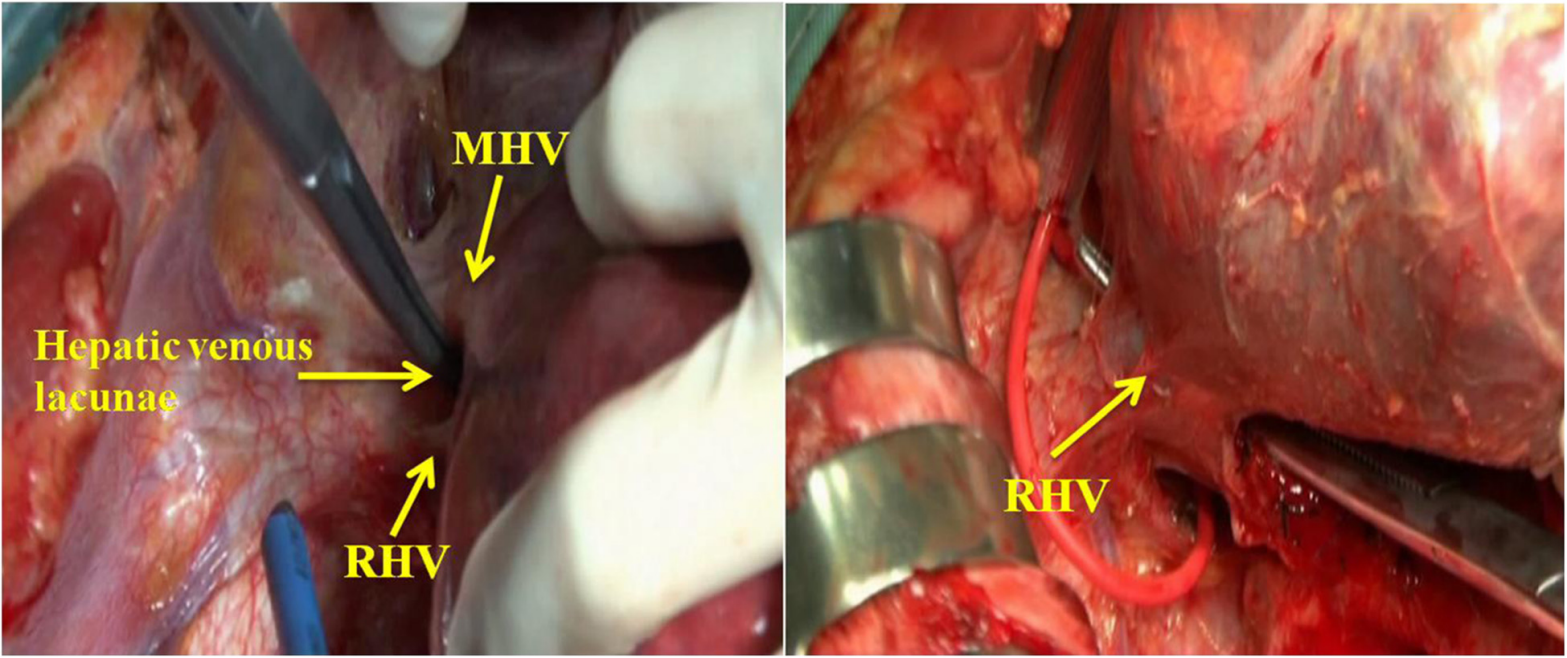
Blunt dissection was used to develop the tunnel before a tape was passed through.

**Figure 6:**
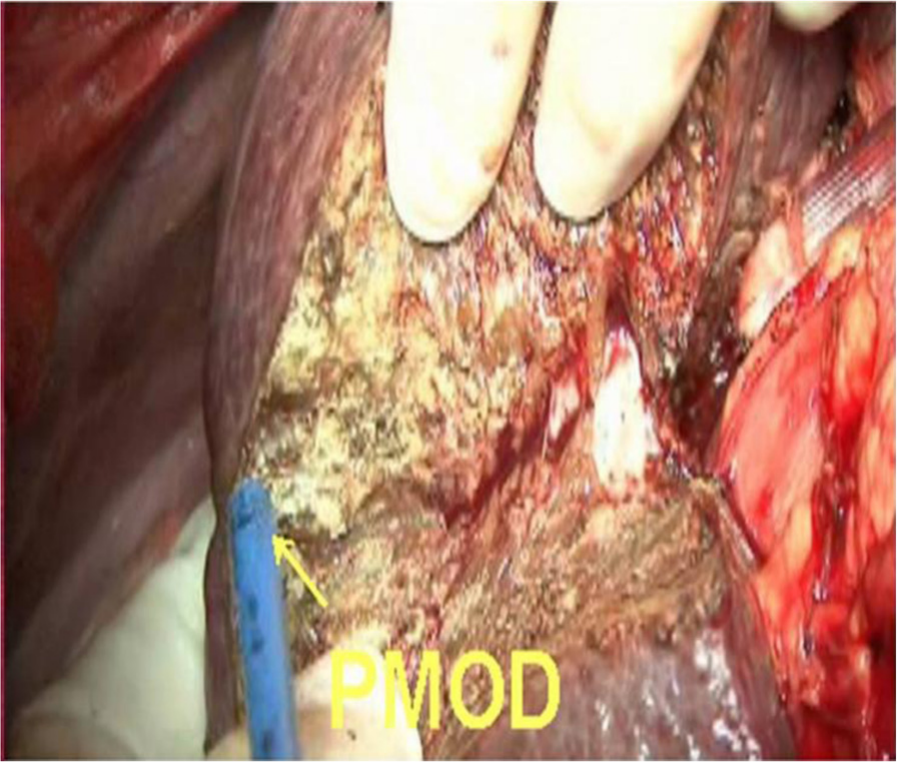
The parenchymal has been transected by Peng’s multifunction operative dissector (PMOD) and the curettage and aspiration dissection technique (CADT).

**Figure 7:**
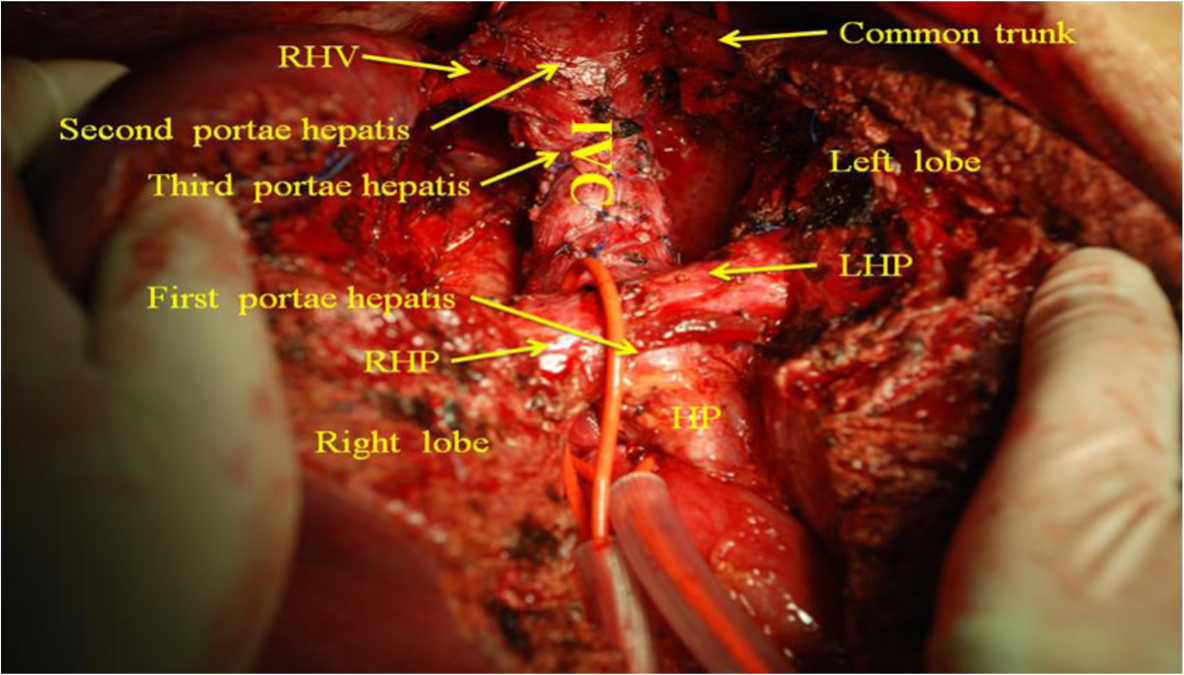
**The liver was split into two halves through the midplane, thus the caudate lobe as well as the first, second, third *portae hepatis *were fully exposed.**

Isolated complete caudate lobectomy using the anterior transhepatic approach included six steps:

1. Liver mobilization: the *ligamentum teres hepatis* was ligated and the falciform ligament was incised from the anterior abdominal wall to the front of the suprahepatic inferior *vena cava* (SIVC). Then the roots of the major hepatic veins were exposed. After the lesser omentum was incised, both the left Spiegel lobe and the left side of the SIVC would be exposed. The incision was made to the right, and the right coronary ligament, right triangular ligament, and hepatorenal ligament were dissected.

2. Blood flow control: the retroperitoneum overlying the infrahepatic inferior *vena cava* (IIVC) was opened at a position right of the IIVC and 1 to 2 cm above the right renal vein. Then the surgeon passed his left index finger behind the IIVC to the left side and guided a tape to encircle the IIVC (Figure 2). The SIVC was dissected from its posterior structure and a clamp was passed through the tunnel behind it toward the left side, then the surgeon encircled the SIVC with tape (Figure 3). Finally, the hepatoduodenal ligament was mobilized and encircled with tape (Figure 4).

3. Short hepatic vein ligation: three to five thick, short hepatic veins were separated in this process. Blunt dissection was used to develop the tunnel before a tape was passed through (Figure 5).

4. Liver-splitting anterior approach: the interlobar plane had been split before the anterior surface of the paracaval portion and the hilar plate were explored.

5. Caudate portal triad ligation: there were three to five caudate portal triads branching from the left and right hepatic pedicle junction into the caudate lobe.

6. Detachment of the caudate lobe from the eighboring liver parenchyma. We used PMOD to transect the liver parenchyma by means of the CADT when intermittent inflow was occlusive (Figures 6 and 7). Tapes were used to encircle the IIVC and hepatic pedicle. The time limit was ten minutes each time with reperfusion for two minutes.

## Results

There were 6 cases with hepatocarcinoma (HCC) associated with liver cirrhosis, 2 cases with large cavernous hemangioma and 1 case with large intrahepatic cholangiocacinoma. All patients had liver function in Child-Pugh A class. Liver hanging maneuver was performed successfully in 8 patients. The median tumor size was 6.2±2.7 cm (range 2.1-10.5 cm). The median operating time was 230±43.6 min (range 170–300 min). The median intraoperative blood loss was 606.6±266.3 ml (range 350–1200 ml). All patients received R0 resections. The median length of postoperative hospital stay was 12.6±2.9 days. Two complications were observed in two patients, including ascits in one patient and bile leakage in another. Both of them were successfully managed medically. There were no perioperative deaths (Table 1).

**Table 1:** General data from the nine patients

**Patient**	**Age/sex**	**Tumor size (cm)**	**Pathology**	**Operating time (minutes)**	**Blood loss (ml)**	**Postoperative** **hospital stay (days)**
1	45/M	5.2	Hepatocarcinoma	210	600	14
2	48/F	7.9	Hemangioma	255	500	12
3	55/F	6.2	Hepatocarcinoma	240	800	10
4	52/M	7.4	Hholangiocacinoma	270	1,200	8
5	60/F	2.1	Hepatocarcinoma	170	450	18
6	65/M	10.5	Hemangioma	260	700	11
7	62/M	3.2	Hepatocarcinoma	205	400	13
8	70/F	8.6	Hepatocarcinoma	300	450	12
9	56/M	4.3	Hepatocarcinoma	180	350	15
All patients (mean ± SD)	57 ± 8.1	6.2 ± 2.7	NA	230 ± 43.6	606.6 ± 266.3	12.56 ± 2.9

Quantitative data are presented as absolute values for individual patients unless stated otherwise. *M*, Male; *F*, Female; *NA*, Not applicable.

## Discussion

Removal of excess liver tissue is not allowed in the process of the hepatic caudate lobectomy, because in China 85% of cases of hepatocarcinoma (HCC) are complicated by cirrhosis. So, isolated resection of the hepatic lobe plays an irreplaceable role in surgical treatment of hepatic tumor. Both left- and right-side approaches are used to resect the caudate lobe when the tumor is small. But when the tumor is large and compresses major hepatic veins, or when cirrhosis is very serious, the above methods cannot be performed because of possible injury to the major hepatic veins [3]. Based on these circumstances, the anterior transhepatic approach is the best choice for isolated complete caudate lobectomy, because it maximizes the exposure of the operative field, and minimizes the operative risks.

Liver resection may be complex due to prolonged operating times and intraoperative bleeding, especially during the separation of the hepatic parenchyma and the resection of lesions close to major hepatic veins, in which unpredictable hemorrhage can be life-threatening [4,5]. PMOD combines four different functions in one: electro-cutting, electro-coagulating, curetting and aspirating [6]. The 40 to 60 HZ power would be sufficient for separation of the hepatic ligament, and the maximum power of 120 HZ can be used for transection of the hepatic parenchyma [7]. We mainly use it to curette on the liver incision line for parenchymal transection. When large vessels are seen, curettage is applied to separate the parenchyma from the vessels in the same layer. Then the vessels can be ligated and dissected in direct view. Therefore, sudden and massive bleeding rarely occurs. Both operating times and hemorrhage are thereby reduced remarkably to make the operation safer. Thus, in our study, the mean operating time and blood loss were 232.2 minutes and 606.6 ml respectively, which are lower than in other reports [8-12].

It has been widely proposed to use hepatic vascular control in hepatic caudate lobectomy [5], but some authors regard it as unnecessary [13]. Low, central venous pressure (CVP) can decrease the pressure in the hepatic veins and hepatic sinusoid, thereby reducing bleeding from these locations [14]. If tapes or blood control in the IIVC are used, CVP could be decreased by 4 to 6 cm H_2_O (1 cm H_2_O = 0.098 kPa) [8]. It was also reported that during surgery, the blood control in the IIVC had the same effect as hypotensive anesthesia in reducing CVP [15]. If the hepatic pedicle and the IIVC are both encircled, bleeding of the hepatic cutting surface will be reduced significantly. If the tumor is so large that it is difficult to implement the IVC blood control for precaution, the surgeon should not try to use tape to encircle the SIVC. However, tumors adhering to the IIVC need to be mobilized to free the IVC to the renal vein, then the tape can encircle the SIVC.

Blunt dissection is used to make the tunnel before the tape is pulled through [16]. A hemostatic plate may be placed on the surface of liver parenchyma if needed. In hepatectomy, the tape is pulled up to create an interspace between liver parenchyma and the IVC so the IVC can be protected during transection. With this method, we can separate the liver in the possible shortest time, and the surgical risk will be reduced to a minimum. Only when the IVC is invaded by the tumor can the liver-hanging maneuver not be performed.

## Conclusions

The anterior transhepatic approach in isolated complete caudate lobectomy is a curative procedure for the tumor located in caudate 1obe, especially suitable for cases with large tumor, cirrhosis and IVC invasion. The application of anterior approach for isolated caudate lobectomy can converse the results of certain kind of caudate lobe tumors from non-resectable to respectable due to widening the indication. The intraoperative routine use of PMOD, application of inflow and outflow of hepatic vascular control, low central venous pressure and selective use of liver hanging maneuver together make the anterior transhepatic approach for isolated complete caudate lobectomy safer and easier.

## Consent

Written informed consent was obtained from the patients for publication of this report and any accompanying images.

## Abbreviations

CADT: Curettation and aspiration technique; CPT: Caudate portal triads; CVP: Central venous pressure; HCC: Hepatocarcinoma; IIVC: Infrahepatic inferior *vena cava*; IVC: Inferior vena cava; PMOD: Peng’s multifunction operative dissector; SIVC: Suprahepatic inferior *vena cava* .

## Competing interests

The authors declare that they have no competing interests.

## Authors’ contributions

YJH, GJ, DP, CL, WWG, MJS, LML, WXS, ZYL, ZL, WH, and DQ designed and conducted the study, analyzed the data, and helped to write the manuscript. LYB is the principal investigator, and revised and edited the manuscript. All authors approved the final manuscript.
